# Rapid Prototyping Flexible Capacitive Pressure Sensors Based on Porous Electrodes

**DOI:** 10.3390/bios13050546

**Published:** 2023-05-14

**Authors:** Tiancong Zhao, Huichao Zhu, Hangyu Zhang

**Affiliations:** 1 School of Biomedical Engineering, Faculty of Medicine, Dalian University of Technology, Dalian 116024, China; zhaotiancong163@163.com; 2 Liaoning Key Lab of Integrated Circuit and Biomedical Electronic System, Dalian University of Technology, Dalian 116024, China; zhuhuichao@dlut.edu.cn; 3 School of Artificial Intelligence, Dalian University of Technology, Dalian 116024, China

**Keywords:** laser-induced graphene, flexible pressure sensor, double-sided engraving, porous electrode, capacitive

## Abstract

Flexible pressure sensors are widely applied in tactile perception, fingerprint recognition, medical monitoring, human–machine interfaces, and the Internet of Things. Among them, flexible capacitive pressure sensors have the advantages of low energy consumption, slight signal drift, and high response repeatability. However, current research on flexible capacitive pressure sensors focuses on optimizing the dielectric layer for improved sensitivity and pressure response range. Moreover, complicated and time-consuming fabrication methods are commonly applied to generate microstructure dielectric layers. Here, we propose a rapid and straightforward fabrication approach to prototyping flexible capacitive pressure sensors based on porous electrodes. Laser-induced graphene (LIG) is produced on both sides of the polyimide paper, resulting in paired compressible electrodes with 3D porous structures. When the elastic LIG electrodes are compressed, the effective electrode area, the relative distance between electrodes, and the dielectric property vary accordingly, thereby generating a sensitive pressure sensor in a relatively large working range (0–9.6 kPa). The sensitivity of the sensor is up to 7.71%/kPa^−1^, and it can detect pressure as small as 10 Pa. The simple and robust structure allows the sensor to produce quick and repeatable responses. Our pressure sensor exhibits broad potential in practical applications in health monitoring, given its outstanding comprehensive performance combined with its simple and quick fabrication method.

## 1. Introduction

Flexible pressure sensors show potential for broad applications, such as electronic skin, healthcare, motion monitoring, intelligent textiles, and aerospace. Constructed of flexible materials, they have outstanding flexibility, ductility, and even the characteristics of free bending and folding, accommodating the demand for the unconstrained placement of the sensors according to the requirements of measurement conditions and complicated scenarios [1,2,3]. The current flexible pressure sensors are classified according to the working principle, including resistance, capacitance, piezoelectric, and triboelectric types [4,5,6]. Capacitive pressure sensors are widely used in research because of their simple structure, signal stability, and low power consumption.

In recent years, numerous novel techniques for flexible pressure sensors have emerged, showing the prospect of their practical application. Some new technologies proposed include improvements in electrode materials and dielectric layer processes. Generally, micropatterning techniques, including photolithography, magnetron sputtering, plasma etching, and freeze drying, are applied to fabricate sensitive elements with micro-/nanostructures [7,8,9,10]. Typical methods include creating microstructures on the surface of a dielectric layer, such as microstructured dielectric polydimethylsiloxane, polyvinylidene fluoride, or graphene oxide. Additionally, pressure sensors could also be integrated with flexible electrodes, such as silver nanowires or graphene, which significantly improves the operational performance of the sensors and generates a series of highly sensitive flexible pressure sensors [11,12,13]. However, typical issues, including the sophisticated and time-consuming fabrication process, the high cost of the technique, and the unsatisfactory stability and robustness of the sensors, may impede the application of these sensors in the real world. In addition, electrodes in traditional capacitive sensors are generally planar electrodes, typically made of metals or carbon [14,15]. The design of such sensors highly relies on the deformation ability of the dielectric layer, whereas electrodes contribute little to the working mechanism of the sensor. Several three-dimensional (3D) electrodes have been proposed in this respect, such as foam metal and microstructured conductive silicone rubber [16,17]. Nevertheless, making these electrodes is expensive and time consuming, and they are not soft enough to respond to subtle pressure.

Here, we propose an ultrathin laser-induced graphene (LIG)-based flexible capacitive pressure sensor with an extremely straightforward and fast fabrication approach. The laser engraves both sides of the polyimide (PI) paper to transform the relative area into LIG electrodes, leaving a thin dielectric layer of PI paper in between [18,19,20]. After coating the thermoplastic polyurethane (TPU) film onto LIG electrodes, the pressure sensor is ready to use, and the fabrication only takes several minutes. The PI paper and TPU film used are relatively low in price. The unique structure and properties make the LIG layers serve as both the electrodes and parts of the dielectric layers. The simple but robust structure leads to a relatively large operational range from 0 Pa to 9.6 kPa and excellent operational stability during 1000 cycles of loading and unloading the pressure. This fast and precise laser-assisted method guarantees the robust operational performance of the ultrathin flexible sensor for practical applications and can be scaled up at a low cost.

## 2. Materials and Methods

### 2.1. The Sensor Fabrication

**Figure 1:**
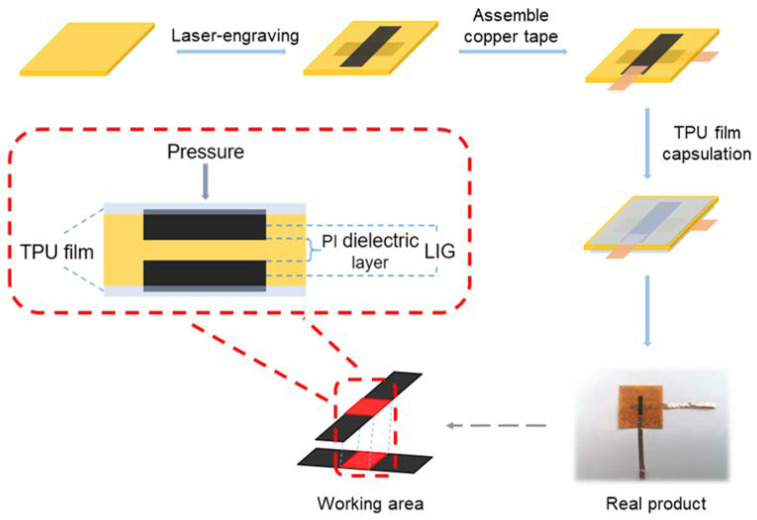
The preparation process of the pressure sensor.

The schematic illustration of the flexible capacitive pressure sensor is shown in Figure 1. Briefly, a laser engraving machine (LaserPecker 2) with a 450 nm diode laser with the output power up to 5 W and a spot size of 50 μm was used to generate LIG electrodes (2 mm × 10 mm) on both sides of a piece of PI paper with a thickness of 90 μm. The LIG electrodes were produced with an optimized power (9%), depth (8%), and 2K resolution. The double-sided conductive copper tape was bonded to the end of LIG electrodes to connect the sensor to the data acquisition device. The material used for electrode capsulation is TPU film. The LIG electrodes were sealed with TPU film with a thickness of 30 μm by hot stamping.

### 2.2. Characterization

A TESCAN Mira3-LMH microscope operating at 10 keV was used to characterize LIG electrode morphology. A Raman analysis was conducted on a LabRAM HR Evolution confocal Raman microscope from HORIBA Scientific and the excitation laser wavelength was set to 532 nm. A portable precision resistance/capacitance measuring device (TruEbox 01RC, LinkZill, Hangzhou, China) was applied to collect capacitance data and operated at a frequency of 250 kHz. The pressure test was conducted with a universal testing machine (ZQ-990B, Zhiqu Precision Instruments, Dongguan, China) or with hands by placing homemade cardboard balance weights onto the sensor. When using the universal machine, the moving speed of the compression head was set to 1 mm/s, and the termination condition was that the pressure reached the set level. The pressure was set to 0.002 N, 0.003 N, 0.006 N, 0.009 N, and 0.003 N/step between 0.012 N and 0.03 N.

### 2.3. COMSOL Simulation of the Electric Field Distribution

The electric field distribution in the pressure sensor was simulated using the finite element software COMSOL Multiphysics 6.0. A simplified honeycomb structure was constructed as the physical model of the porous LIG electrode and the relevant parameters of the physical mode were set up with the consideration of the actual size. The rectangular part in the middle represents the thin layer of residual PI paper between LIG electrodes, and the porous structure on both sides represents the LIG electrodes. A finite element analysis was performed for the electric field intensity distribution of the electricity module. The frequency was set to 250 kHz to correlate with the frequency of the capacitance measuring device. The lower electrode was connected to ground, while the lower surface potential of the other electrode was set as 1 V.

## 3. Results and Discussion

**Figure 2:**
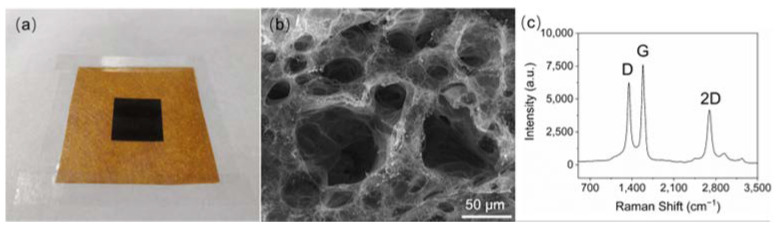
The general characterization of LIG. (**a**) A photo of LIG on the PI paper. (**b**) The porous network structure of LIG. (**c**) The Raman spectrum of LIG.

The LIG-based pressure sensor was fabricated by laser engraving the PI paper on both sides to generate two LIG electrodes with a thin layer of residual PI paper in between as the dielectric layer. LIG technology utilizes appropriate laser engraving parameters to convert polymer substrates into conductive LIG materials with 3D structures based on photothermal carbonization, providing a fast, facile, and low-cost fabrication method to construct porous conductive materials [18,19,20]. As demonstrated in Figure 1, there are only a few simple steps in the fabrication process that take only several minutes without sophisticated instruments or expensive materials. In agreement with previous reports, a typical 3D porous LIG structure is obtained as illustrated in the SEM image and the Raman spectrum (Figure 2) [21,22,23]. The sp^2^ carbon signature of graphene is characterized by the G peak at around 1580 cm^−1^. The defect structure of LIG and the bending of the sp^2^ carbon leads to the appearance of the LIG-specific D peak at 1350 cm^−1^, whereas the presence of 2D peaks around 2700 cm^−1^ accounts for the second-order zone boundary phonons. The sensor is connected to a portable meter controlled by a smartphone via Bluetooth.

**Figure 3:**
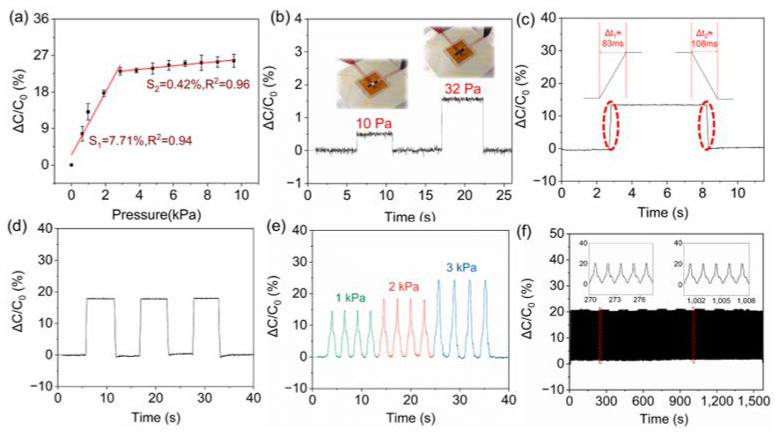
The operational performance of the sensor. (**a**) Sensitivity test. Real-time monitoring of quick response to slight pressures (**b**), loading/holding/unloading cycles of a homemade cardboard balance weight (**d**), pressure pulses (**e**), and 1000 loading/unloading cycles displaying operational stability (**f**). (**c**) Response to pressure showing response and relaxation time.

In order to characterize the performance of the capacitive pressure sensor based on double-sided engraving, a computer-controlled universal testing machine is used to apply pressures to the sensor with a 2 mm × 2 mm working region defined by the overlapping area of two electrodes. The capacitive responses to the pressures are recorded and displayed in Figure 3a. It can be seen that the sensor responds to the pressures in a relatively broad range from 0 Pa to 9.6 kPa in a segmented regression, which is common for pressure sensors [24,25,26]. In the low-pressure range (0~2.865 kPa), the sensor exhibited a relatively high sensitivity of 7.71% kPa^−1^. With the increase in pressure, the deformation state of the 3D network structure of LIG tends to be saturated, resulting in a relatively low sensitivity of 0.42% kPa^−1^ between 2.865 kPa and 9.6 kPa. Hand-placing homemade cardboard balance weights demonstrates the sensor responses to the subtle pressure and response speed. Discernible capacitance increments were observed upon dropping light weights that generated subtle pressures of 10 Pa and 32 Pa, as shown in Figure 3b. The simple and robust structure as well as the excellent mechanical elasticity of LIG allow the sensor to produce quick and repeatable responses. The response and relaxation times are around 100 ms, making the sensor suitable for relatively high-frequency pressure stimulation (Figure 3c). In addition to the quick response, the signal is stable and repeatable without any fluctuation when holding the pressure on the sensor (Figure 3d). Figure 3e displays the repeated responses of our sensor upon loading and unloading various pressure pulses from 1 kPa to 3 kPa. Additionally, the operational stability under repeated loading was examined using the computer-controlled universal testing machine at a frequency of 0.67 Hz (Figure 3f). Negligible fluctuation was observed during the 1000 cycles, exhibiting the excellent durability and repeatability of the sensor.

**Figure 4:**
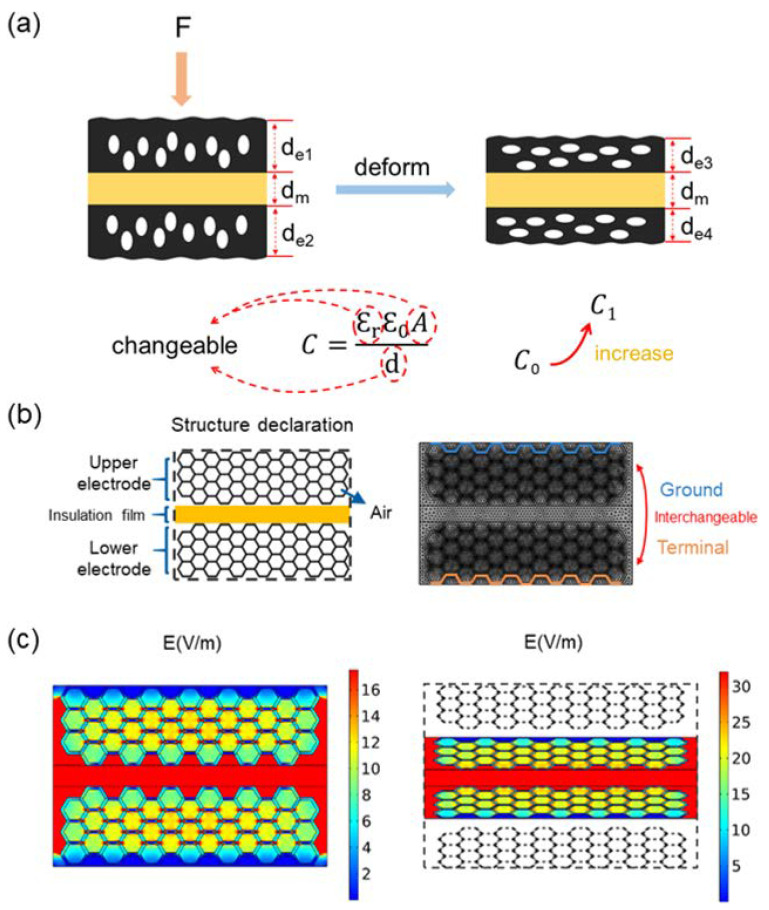
Analysis of the sensor working principle. (**a**) The diagram shows how the sensor works. (**b**) The simulation model and grid diagram of electric field division. (**c**) COMSOL simulation results of the initial state (**left**) and the final state (**right**).

The working principle of the sensor is illustrated in Figure 4. LIG formed by laser engraving has an elastic porous structure. When the porous structure is compressed, the relative distance between electrodes decreases, the effective electrode area of the sensor increases, and the overall relative dielectric constant of LIG alters due to the squeezing out of the air inside the porous structure. According to the calculation formula of parallel plate capacitance (Figure 4a), any parameter changes impact the capacitance [25,27,28]. When using metal electrodes, the charges are primarily distributed on the electrode surfaces approaching the insulated layer. Nevertheless, LIG is not as conductive as metals, leading to the electric field distribution inside the electrodes, which is verified by the electric field distribution simulation experiment using the finite element analysis software COMSOL [29]. As shown in Figure 4b,c, the simulation model was appropriately constructed and assigned experimental settings. A relatively weak electric field is distributed inside LIG electrodes compared with the dense distribution between two electrodes. In this case, LIG electrodes also behave as a dielectric structure, and the interior electric field distribution alters considerably as the sensor is compressed. Unlike the commonly applied porous structure within the dielectric layer or air chambers constructed by micropillars, the LIG electrodes work as the pressure-absorbing component and generate the capacitance variation under compression. The LIG-based pressure sensor fabricated with the double-sided laser engraving approach proposed in this work is competitive in all-around performance compared with recent research on porous dielectric layers or electrodes (Table 1). Most studies focus on microstructured dielectric layers, with little research on porous electrodes. Complicated and time-consuming processing methods are generally applied in the reports on porous electrodes [29,30]. Therefore, the sensor proposed in this study has unique advantages regarding the fabrication process.

**Figure 5:**
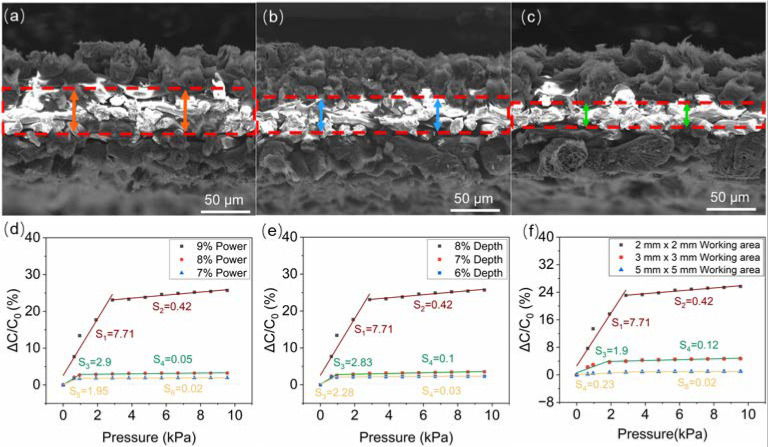
Parameters that influence the sensitivity. (**a**–**c**) SEM images showing the dielectric layer (indicated with red dashed rectangles) of different thicknesses generated with varied laser engraving parameters. Adjustment of the sensitivity with varied laser power (**d**), depth (**e**), and size of LIG electrodes (**f**).

The thicknesses of the LIG electrodes and the dielectric layer can be simply controlled by adjusting the laser-engraving parameters. As the laser power and engraving depth comprehensively affect the laser engraving outcome, the greater the laser power and the engraving depth, the thicker the LIG layer, illustrated by SEM profiles (Figure 5a–c). The rough boundaries between LIG and PI are indicated with red dashed lines. Sensors with thicker elastic LIG electrodes and a thinner incompressible PI layer have a greater sensitivity and working range (Figure 5d,e). As discussed above, LIG electrodes also behave as a dielectric structure, which, together with the residual PI paper between LIG electrodes, form the overall dielectric layer. Assuming Young’s modulus of LIG electrodes with different thicknesses is the same, the strains of LIG electrodes with different thicknesses are the same under a certain pressure. Then, the deformation distance is larger in the thicker electrode. According to the capacitance formula in Figure 4a, the variation in d is greater for the sensor with thicker LIG electrodes under the same pressure, resulting in a more significant change in the capacitance and higher sensitivity. When the engraving parameters are fixed, a sensor with a smaller working area has a smaller initial capacitance (C_0_), which is beneficial for greater sensitivity (Figure 5f). When the working area increases, the complex phase shift of the sensor signal also increases under the excitation of the alternating current module of the RC measuring device, which significantly lowers the sensitivity. Consequently, the sensitivity of the sensor is influenced by laser-engraving parameters and the physical size of the LIG electrodes.

**Figure 6:**
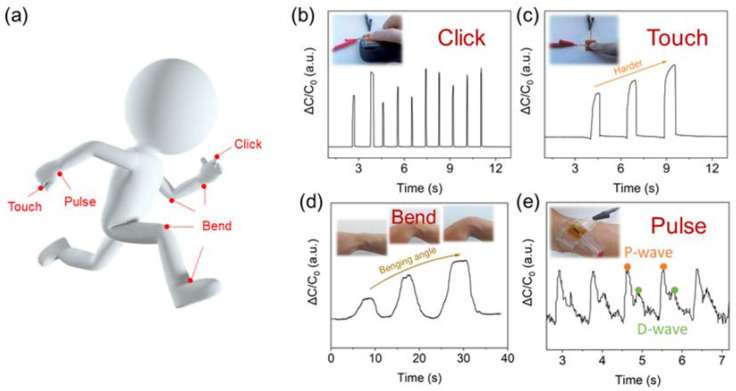
Practical applications of the pressure sensor. (**a**) Illustration of the position for the sensor to measure the pressure. Measurement of the pressure signal engendered by the click of the mouse (**b**), fingertip touch (**c**), wrist bending (**d**), and pulse (**e**).

In recent reports, pressure sensors have been used to detect human behavior signals such as walking, grabbing, and touching or human medical signs such as pulse [11,37,38]. The rapid prototyping flexible pressure sensor with a simple and robust structure designed in this study can be applied to various scenes, such as measuring physical signals on various skin positions, as illustrated in Figure 6a. When the sensor was fixed on the mouse surface for the click test (Figure 6b), an apparent increase in capacitance was observed immediately at each click, and the capacitance decreased rapidly as the finger left the surface of the mouse. When touching the sensor on the table, the response signal was positively related to the finger-tapping forces (Figure 6c). Similar results were observed in the bending test, in which the response increased as the bending angle of the wrist became larger (Figure 6d). Finally, the sensor was mounted on the surface of the wrist with tapes to detect the pulse. A typical pulse waveform represented by the P-wave and D-wave was obtained and is displayed in Figure 6e. The characteristic information extracted from the pulse waveform can be used for disease diagnosis. For instance, the ratio of Height 2 (peak of D-wave to baseline) and Height 1 (peak of P-wave to baseline) can reflect the degree of atherosclerotic vessels outside the coronary artery [39].

**Figure 7:**
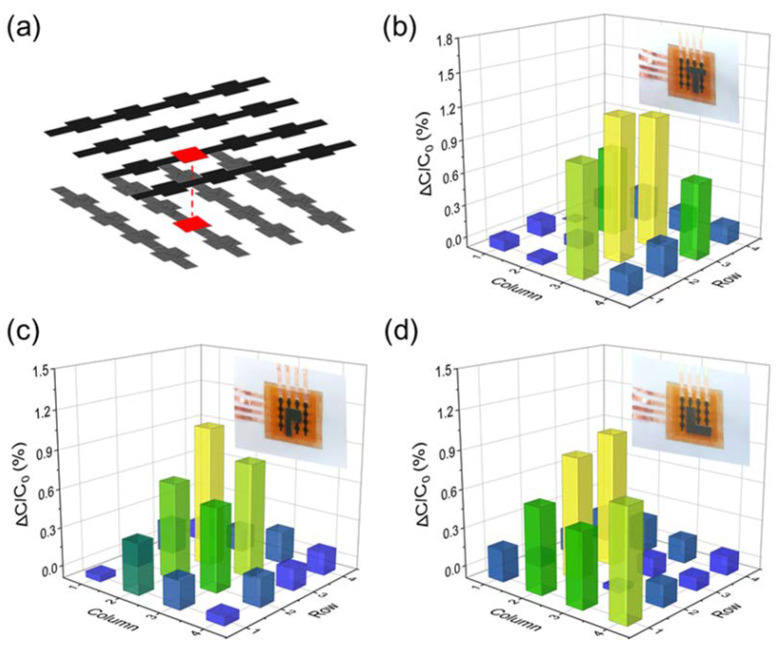
Illustration of a 4 × 4 pressure sensor array constructed for spatial pressure measurement. (**a**) The electrode pattern of the sensor array. Images of homemade T-shaped (**b**), P-shaped (**c**), and L-shaped (**d**) cardboards on the sensor array and the corresponding response on each pixel.

Based on the simple and reliable fabrication of a single pressure sensor, a 4 × 4 pressure sensor array was prepared with the double-sided laser engraving method in several minutes. It turns out that the fabrication approach is easily scaled up. The electrodes and leads were all formed by LIG technology, and the electrode pattern is demonstrated in Figure 7a. Each detection pixel measures 2 mm × 2 mm, and the overall size of the sensor array is within 3 cm × 3 cm. Homemade T-shaped, P-shaped, and L-shaped cardboards were placed on the sensor array to provide position-controllable pressure (Figure 7b–d). When the pressure stimulation is applied to the sensor array, the capacitance of the corresponding detection pixel increases rapidly compared with the rest. Accordingly, the sensor array can be applied for mapping pressure distribution with an acceptable level of crosstalk among neighboring pixels. A relatively low crosstalk in the pressure sensor array is commonly observed, considering the influence of parasitic capacitance and the physical deformation generated from the adjacent capacitors [11,40,41].

## 4. Conclusions

In conclusion, we prepared a flexible capacitive pressure sensor in a double-sided engraving configuration using LIG technology. The unique structure of elastic porous LIG electrodes endows the sensor with high sensitivity, a wide working range, short response/relaxation times, and excellent repeatability and stability without using complicated, time-consuming, and expensive fabrication techniques. The sensor shows the capacity to detect general body movements and pulse. Given its rapid fabrication, ease of scaling up, and robust performance, this sensor has great potential in medical diagnosis and health monitoring.

## Figures and Tables

**Table 1:** (NR = Not reported; DL = Dielectric layer; SR = Silicon rubber; CB = Carbon Black; CNT = Carbon nanotube).

Authors	Feature	Sensitivity	Work Range	Limit of Detection
This work	Porous LIG electrode	0.0771 kPa^−1^	0–9.6 kPa	10 Pa
Jing et al. [30]	Micropatterned porous PDMS (DL)	143.5 MPa^−1^	0.068–150 MPa	68 Pa
Qing et al. [31]	Silicon rubber/NaCl/carbon black (DL)	3.15 kPa^−1^	0–200 kPa	27 Pa
Xue et al. [32]	Porous AgNWs-PDMS (DL)	0.62 kPa^−1^	0–7 kPa	NR
Maeum et al. [33]	CNT-PDMS (DL)	0.056 kPa^−1^	0–110 kPa	NR
Lekshmi et al. [34]	Graphene coated PDMS foam (DL)	0.137 kPa^−1^	0–12 kPa	50 Pa
Yan et al. [35]	CNT-doped porous electrode	1.033 kPa^−1^	0–30 kPa	12 Pa
Long et al. [36]	Conductive coating 3D network electrode	10.2 kPa^−1^	0–100 kPa	0.17 Pa

## Data Availability

Not applicable.
